# Community Awareness of HPV Screening and Vaccination in Odisha

**DOI:** 10.1155/2015/694560

**Published:** 2015-12-09

**Authors:** Niharika Khanna, Aparna Ramaseshan, Stephanie Arnold, Kalpana Panigrahi, Mark D. Macek, Bijaya K. Padhi, Diptirani Samanta, Surendra N. Senapati, Pinaki Panigrahi

**Affiliations:** ^1^Department of Family & Community Medicine, University of Maryland, MD, USA; ^2^Department of Obstetrics and Gynecology, University of Maryland School of Medicine, Baltimore, MD 21201, USA; ^3^Department of Epidemiology, Center for Global Health & Development, College of Public Health, University of Nebraska Medical Center, Omaha, NE, USA; ^4^Department of Dental Public Health, University of Maryland School of Dentistry, Baltimore, MD, USA; ^5^Asian Institute of Public Health, Bhubaneswar, Odisha, India; ^6^Department of Medical Oncology, Acharya Harihar Regional Cancer Center, Cuttack, Odisha, India; ^7^Department of Radiation Oncology, Acharya Harihar Regional Cancer Center, Cuttack, Odisha, India; ^8^Department of Pediatrics, University of Nebraska Medical Center, Omaha, NE, USA

## Abstract

*Introduction*. A number of new technologies including cervical cancer screening and vaccination have introduced new tools in the fight against cervical cancer.* Methods*. This study was set in Odisha, India, at the Acharya Harihar Regional Cancer Center and study research infrastructure at the Asian Institute of Public Health. IRB approvals were obtained and a research assistant recruited 286 women aged 18–49 years, who provided informed consent and completed a survey tool. Data were entered into EpiData software and statistical analysis was conducted.* Results*. 76.3% women participants were married, 45.5% had sexual debut at age 21 or greater, 60.5% used contraception, 12.2% reported having a Pap smear in the past, and 4.9% reported having prior genital warts. Most, 68.8% had never heard of HPV and 11.9% were aware that HPV is the main cause of cervical cancer. 82.9% women thought that vaccinations prevent disease, and 74.8% said they make the decision to vaccinate their children.* Conclusion*. The Odisha community demonstrated a low level of knowledge about cervical cancer prevention, accepted vaccinations in the prevention of disease and screening, and identified mothers/guardians as the key family contacts.

## 1. Introduction

Cervical cancer continues to be a major health problem in India where 132,000 women are diagnosed each year and 74,000 deaths are observed [1]. The majority, 70% of cervical cancers, is detected at Stage III or higher, leading to high mortality rates [2]. Despite an annual cervical cancer incidence rate of 25/100,000 in India, there are no large scale public health surveillance programs in cervical cytological screening and human papillomavirus (HPV) typing in India. Human papillomavirus prophylactic vaccine has been available in the Western world since 2006 due to which there is a real possibility of primary prevention of cervical cancer caused by HPV types 16 and 18.

We designed a study to explore community awareness of HPV and cervical cancer among women in Odisha, India. In addition, we explored the acceptance and understanding of primary and secondary prevention methods for cervical cancer control by adult women who are potential recipients of HPV screening and gatekeepers to their families health. We also explored the perception by women in Odisha to identify the health decision maker for vaccination for young girls in their family. Lastly, we explored possible acceptance of the HPV vaccine and cervical cancer screening in the Odisha community.

This information will inform implementation and dissemination research in cervical cancer prevention including cervical HPV screening and HPV vaccine acceptance.

## 2. Methods

### 2.1. Site

Odisha is a site where organized maternal and child health research has been conducted successfully over the last several years by our collaborating team at the Asian Institute of Public Health [3]. Odisha has a population of 36.8 million inhabitants of which 15% are urban populations [4]. There is access to reproductive age women and organizational capacity for the ethical conduct of human research. In addition, there are 6.1 million inhabitants with less than primary school or no education. There have been no organized government sponsored educational campaigns in this area to increase awareness of HPV and cervical cancer control.

### 2.2. Research Team

There is an existing collaboration arrangement between University of Nebraska Medical Center, College of Public Health, the University of Maryland, the Asian Institute of Public Health (AIPH), and the Acharya Harihar Regional Cancer Center in Cuttack, Odisha. These groups have engaged in collaborative maternal and child health research in these areas and have demonstrated the ability to conduct NIH funded research consistently over the last 12 years. The AIPH utilizes an organized network of 212 villages where there is capability for responsibly conducting research which complies with International Standards of Clinical Research and Harmonization at three levels. There are community health workers known as Anganwadi workers, who are supervised by field supervisors. The field supervisors report directly to the research team at the AIPH. There is capability to ensure that Good Clinical Practices are followed and each Anganwadi worker is trained and retrained in the ethical conduct of human research. Population outreach has been primarily to maternal and child populations over the last 12 years in urban, rural, and tribal settings. In preparation for a future HPV vaccine implementation project, we proposed formative research to sample urban, urban slum, and rural populations in Odisha to approach women and mothers aged 18–49 years to educate and inform them about cervical cancer prevention strategies including cervical cancer screening and the HPV vaccine. We propose to assess their understanding and acceptance of cervical cancer screening and HPV vaccine in the future.

### 2.3. Patient Recruitment

Following Institutional Review Board approvals at both University of Maryland and the Acharya Harihar Regional Cancer Center (AHRCC), we included a total of 286 women aged 18–49 years, who provided informed consent to participate in the study. We excluded those women who had a previous diagnosis of cervical cancer. Women between 18 and 49 years of age who were willing to provide informed consent were included in this study. Women who had a previous diagnosis of cervical cancer were excluded from the study. Women were recruited from urban, rural, and tribal areas to capture the knowledge and attitudes of women in different sociocultural and economic strata. The urban surveys were collected at various local women's colleges, malls, and open markets in Bhubaneswar, the capital city of Odisha. The rural surveys were obtained from the villages in the outskirts of Bhubaneswar. The tribal surveys were collected from the tribal sites in Rourkela, Odisha. A significant number of surveys were obtained from patients visiting the Regional Cancer Center which contained a mixture of women from both urban and rural areas. The participants from the cancer center site included women who were patients, employees, and bystanders at the facility.

### 2.4. Enrollment and Survey Administration

US and AIPH researchers worked together to approach women presenting to the Acharya Harihar Regional Cancer Center, in the urban slums surrounding the AHRCC and the AIPH. Rural women were recruited when presenting to the organized clinical centers and also from the marketplace. Each participant underwent informed consent procedures followed by enrollment procedures including Unique Identifier number assignment. Confidentiality was maintained and each participant was reassured about their anonymity prior to implementing the sensitive research survey tool. The survey was administered by research personnel in the native Oriya language or English and was read to those participants who were illiterate. Answers were recorded in paper format followed by data entry by an assistant into an EpiData database. Data gathering was done with US researchers and AIPH researchers working together to approach women presenting to the Acharya Harihar Regional Cancer Center, in the urban slums surrounding the AHRCC and the AIPH. Rural women were recruited when presenting to the organized clinical centers and also from the marketplace. Each participant underwent informed consent procedures followed by enrollment procedures including Unique Identifier number assignment. Confidentiality was maintained and each participant was reassured about their anonymity prior to implementing the sensitive research survey tool. The survey was administered by research personnel in the native Oriya language or English and was read to those participants who were illiterate.

### 2.5. Data Gathering

It was done by US researchers and AIPH researchers working together to approach women presenting to the Acharya Harihar Regional Cancer Center, in the urban slums surrounding the AHRCC and the AIPH. Rural women were recruited when presenting to the organized clinical centers and also from the marketplace. Each participant underwent informed consent procedures followed by enrollment procedures including Unique Identifier number assignment. Confidentiality was maintained and each participant was reassured about their anonymity prior to implementing the sensitive research survey tool. The survey was administered by research personnel in the native Oriya language or English and was read to those participants who were illiterate. Data gathering was done with US researchers and AIPH researchers working together to approach women presenting to the Acharya Harihar Regional Cancer Center, in the urban slums surrounding the AHRCC and the AIPH. Rural women were recruited when presenting to the organized clinical centers and also from the marketplace. Each participant underwent informed consent procedures followed by enrollment procedures including Unique Identifier number assignment. Confidentiality was maintained and each participant was reassured about their anonymity prior to implementing the sensitive research survey tool. The survey was administered by research personnel in the native Oriya language or English and was read to those participants who were illiterate. When the survey was conducted orally, the answers were recorded on paper by the survey administrator, which was later transferred into EpiData database (version 3.1). Dual database entry and standard data fidelity procedures were followed to ensure accuracy of the data entry and database cleaning. Database access was password protected and access was restricted to key personnel in the Odisha and US team.

### 2.6. Survey Details

The survey was administered by a research assistant who provided translation and helped with completion to those women who requested help. Women were queried in the following domains: demographics, patient knowledge, attitudes and self-efficacy outcomes around vaccination, health care for themselves and their families, HPV knowledge, and acceptability of cervical cancer screening methods and HPV vaccination.

## 3. Statistical Methods

### 3.1. Survey Tool

It is approved by University of Maryland and Local Odisha IRB, administered by a research assistant using the local language and vernacular data management and analysis.

Responses were manually entered into an EpiData software followed by Unique Identifier number assignment to all participants. Data was deidentified and statistical analysis was conducted. Data was systematically analyzed using univariate and bivariate statistical analyses that were conducted using SAS software (Version 9.1) for Windows. Data was analyzed using the following variable groupings: general information, self-efficacy, social supports, and attitudes towards HPV screening and HPV vaccination. Cross-cutting themes and variables were identified and adjusted for cell frequency, percentages, and odds ratios that were calculated by exact procedures available in SAS. All *p* values were computed by Chi-square test for large samples and by Fisher's exact test for small samples. Factors that may influence the acceptance of HPV screening and HPV vaccination included age, marital status, education, smoking status, self-efficacy, social supports, whether prior Pap received, HPV status, and attitudes towards HPV screening and HPV vaccination.

Data were entered into an EpiData database using dual data entry, and over 10% of the database was further audited to ensure accuracy and completeness. Each participant was assigned a unique study identifier number to ensure confidentiality, and the database with names was kept in locked room to which only selected study staff had access. Data were analyzed using SAS (Release 9.1, SAS Institute Inc., Cary, North Carolina, USA). Univariate and multivariate statistical analyses were conducted using SAS software.

## 4. Results

### 4.1. Demographics

This data was gathered in 2009 in the state of Odisha in 286 urban and rural women presenting to the Acharya Harihar Regional Cancer Center for care, from a public marketplace and from a community college for women. There were 286 women who were willing to participate in the survey and demographics are presented in Table 1. We recruited women between the ages of 18 and 49 years and the majority 76.3% were married or formerly married. The majority, 45.5%, had sexual debut at age 21 or greater and 60.5% used contraception. Tobacco use was reported in 13.6% women and 12.2% reported having a Pap smear in the past and 4.9% reported having prior genital warts (Table 1). Most women participants, 68.8%, had never heard of HPV and 11.9% were aware that HPV is the main cause of cervical cancer. Although 46.9% of women know that you might die from cervical cancer only 15% believed that their current physician would take care of their cervical cancer needs. The understanding that vaccinations prevent disease was widely prevalent and 82.9% of women thought that vaccinations prevent disease, and 74.8% thought that they would be the person in their family to make the decision to vaccinate their children. There was less clarity on whether their community would resist the HPV vaccine and 9.1% of women clearly thought there would be resistance, whereas 29.7% did not know and 7.3% did not answer this question (Table 2).

**Figure 1:**
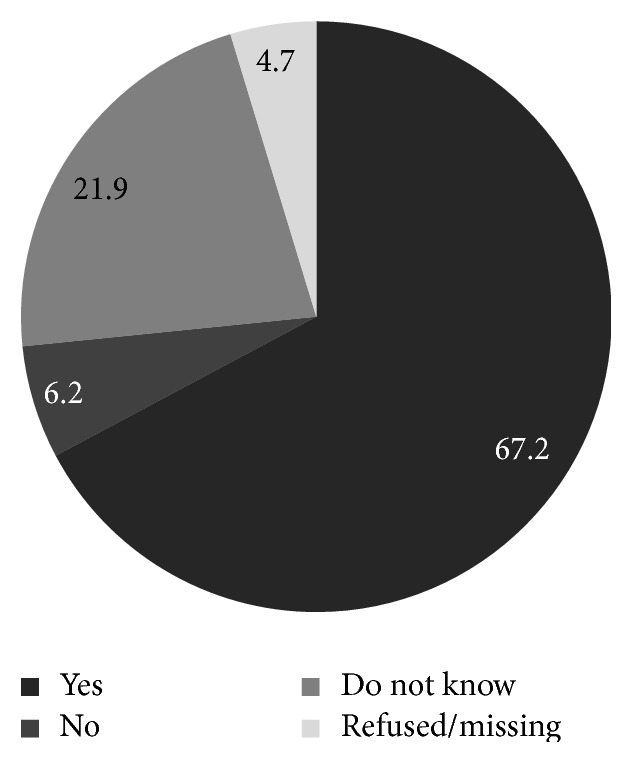
Aware that HPV results from sex with infected partner, among those who have heard of HPV, Odisha, India (*n* = 64).

**Figure 2:**
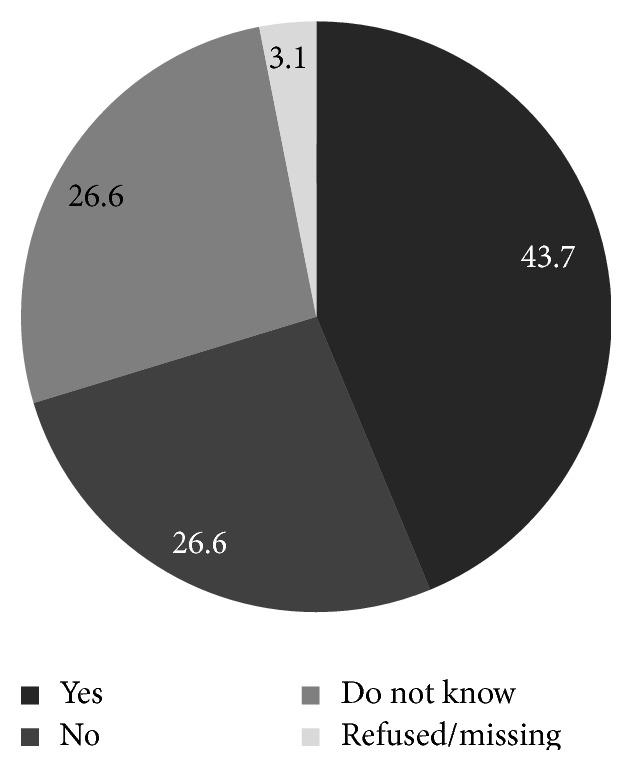
Aware that HPV is the main cause of cervical cancer, among those have heard of HPV, Odisha, India (*n* = 64).

**Figure 3:**
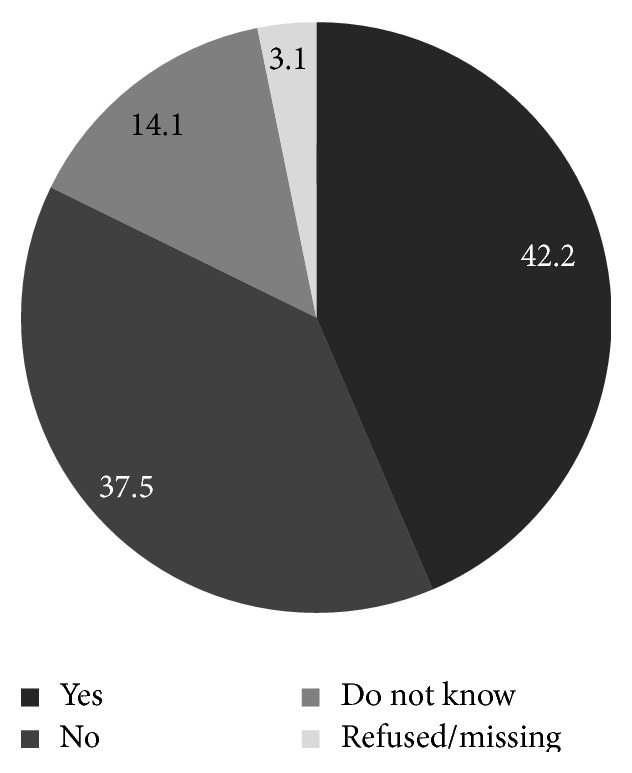
Aware that cervical cancer can be prevented by an HPV vaccine, among those who have heard of HPV, Odisha, India (*n* = 64).

Among the women who had heard of HPV, women aged 20–29 years were most likely to have heard of HPV and those older than 40 years were least likely to have heard of HPV. In addition, those who had heard of HPV were more likely to be single and to have never had sex (Table 3). Among the 64 women who were aware of HPV, 67.2% stated awareness of the sexual transmission of HPV through an infected partner (Figure 1), 43.7% had the knowledge that HPV is the main cause of cervical cancer (Figure 2). Among these 64 women, only 42.2% were aware that cervical cancer prevention is feasible with the novel HPV vaccine and 37.5% women stated that they were unaware that vaccine mediated prevention was feasible (Figure 3).

## 5. Discussion

Cervical cancer is an important disease in Odisha and public health campaigns have not occurred to increase community awareness. Recent developments linking HPV to cervical cancer are not known in the community and there is no existing concerted effort to develop public health campaigns to increase this awareness. Community awareness and buy-in is critical to the introduction of newer strategies for cervical cancer prevention [5]. Recent events in India have led to governmental review of policies surrounding HPV vaccine clinical trials in India [6–8]. A key point raised by activists and others is the vulnerability of the targeted population and the need for review of the ethical mandates by governmental agencies. There is no doubt that affordable, safe vaccination could significantly impact the incidence and mortality from cervical cancer in India. However, community awareness, health education, HPV vaccine, and a complementary robust screening program are essential for appropriate cervical cancer impact. Introduction of the HPV vaccine in exploratory studies of acceptance, safety, and immunogenicity in the Indian population are necessary before any consideration for government policy and development of cervical cancer prevention programs. Evaluation of acceptance by the community is necessary prior to the consideration of such clinical implementation studies [9].

The implementation of the HPV vaccine in Western countries has been surrounded by sociocultural issues raised by communities and in several countries, there has been no inclusion in government sponsored and mandated vaccination programs [10]. By extension of this concept, it is expected that there will be significant challenges to the introduction of the HPV vaccine in India where there is low awareness of human papillomavirus and link to cervical cancer. Further, there are challenges with the introduction of a sexually charged discussion with mothers of girls aged 10–14 years. In addition to cultural issues, religion and the impact of public opinion are critical to the understanding of future methods of HPV vaccine implementation in India.

In developed countries, cervical cancer incidence and mortality have been significantly reduced following the introduction of screening and early detection using cytology. In the US, cytological screening was introduced using public health campaigns led by the American Cancer Society in the 1950s and steady decline in incidence has been observed [11]. Methods for screening were further refined using HPV typing which was introduced to practicing physicians in 1999 in the US. These introductions of new screening and early detection methodologies are accompanied by multimedia campaigns and grassroots advocacy to achieve universal acceptance by the physician and patient communities. Similarly, the introduction of the novel HPV vaccine is accompanied by multimedia campaigns, government policy supporting primary prevention, and the implementation of clinical procedures which allow for increased penetration of primary prevention into the developed world. Newer cervical cancer prevention strategies in the US are based on the potential for primary and secondary prevention using screening and treatment for women who are already infected with HPV and primary prevention using the HPV vaccine for girls in these same communities. This is the first cancer prevention strategy that utilizes robust interventions at the primary and secondary prevention levels and these strategies have enormous potential to reduce the burden of cervical cancer in areas with high incidence [12].

Utilizing the Western experience to extrapolate the understanding of cervical disease prevention to a medium resource country such as India, selecting a strategy that is universally accepted and has high effectiveness will demonstrate the greatest success in the reduction of cervical cancer. Thus, both cervical cancer screening and vaccination have the potential for success in decreasing the incidence, the morbidity, and mortality from cervical cancer. Further, these combined strategies will for the first time allow a shift in detection of advanced stages of cervical cancer to early detection of cervical precancer and reduce the development of cervical disease in girls who receive the HPV vaccine. Considering the advantages and disadvantages of both these strategies individually and collectively will lead to the highest likelihood of success. It is critically important to assess community awareness and sensibilities, combined with good science to guide the development of advocacy and policy which determines the most efficient methods applicable to India. Further, it is important to develop rigorous clinical implementation projects which are limited to a selected region of the country and focus on detailed information gathering which can then form the basis for informed decision making for communities and for governmental agencies [13].

Our Odisha data effectively produced a window into the selected community. Odisha is a site where there have been no government or industry sponsored campaigns to increase community level awareness of cervical cancer prevention and HPV. Thus, our project has the advantage of assessing a community prior to the introduction of educational processes to enhance community awareness and buy-in to cervical cancer prevention methods. Our project included a wide spectrum of women, from those presenting to the regional cancer center, and thus an already aware population, and women from the marketplace who formed a less targeted population. We also recruited women at the local government college where we were interested in the views of young educated women. Thus, the data presented here represents the opinions of a wide variety of women from the State of Odisha.

There was very low awareness of cervical cancer, of cervical cancer prevention, and of HPV and the link to cervical cancer. Interestingly, 12.2% of women had received the Pap smear and clearly there is scope in the rest of the interviewed women to increase the number of women screened by either cytology or HPV typing to ensure cervical cancer secondary prevention. There appeared great interest in disease prevention and our team assessed and were impressed by the proactive stance presented by women as an indicator of future receptivity to education strategies to enhance community awareness leading ultimately to acceptance of dual primary and secondary cervical cancer prevention strategies. There was high awareness of the value of vaccines in preventing disease and women interviewed were confident that they would make the decision for their families to vaccinate against disease. This is borne out in prior research, where women are known gatekeepers of the health of their families and make health related decisions for their family units. This also suggests that future educational campaigns for cervical cancer prevention should target women as recipients of health education and the development of decision support tools for women regarding cervical cancer screening and vaccination [14]. Cervical cancer reduction in India will reduce one quarter of the global burden of disease and impact the global community. It is encouraging that GAVI (The Vaccine Alliance) is considering the HPV vaccine for delivery to underserved populations globally and that discussions are ongoing. In order to decrease the incidence and ultimately to eliminate cervical cancer, new innovations such as the HPV vaccine have enormous potential for success in reducing global burden of disease.

## 6. Conclusion

The Odisha community surveyed demonstrated an acceptance of screening and vaccinations in the prevention of disease and identified mothers/guardians as the key family contacts. There is an opportunity to increase their knowledge of human papillomavirus, its link to cervical cancer, and the ability of the HPV screening and vaccine to prevent cervical cancer. This opportunity should be utilized to outreach and develop community awareness and encourage acceptance and adherence to three doses of the HPV vaccine and HPV screening to reduce cervical cancer.

## Figures and Tables

**Table 1:** Sample characteristics, Odisha, India (*n* = 286).

Characteristic	Sample size	Percentage
Age		
20–29 years	98	34.3
30–39 years	104	36.3
≥40 years	80	28.0
Refused/missing	4	1.4
Marital status		
Single (never married)	67	23.4
Married/formerly married	218	76.3
Refused/missing	1	0.3
Sexual history		
Never had sex	52	18.2
First sex at <21 years	85	29.7
First sex at ≥21 years	130	45.5
Refused/missing	19	6.6
Ever used contraception		
Yes	173	60.5
No	113	39.5
Ever used tobacco products		
Yes	39	13.6
No	241	84.3
Refused/missing	6	2.1
Ever had genital warts		
Yes	14	4.9
No	240	83.9
Do not know	19	6.6
Refused/missing	13	4.6
Ever had a Pap smear		
Yes	35	12.2
No	204	71.3
Do not know	24	8.4
Refused/missing	23	8.1

**Table 2:** Knowledge, attitudes, and self-efficacy outcomes, Odisha, India (*n* = 286).

Characteristic	Sample size	Percentage
Believe cervical cancer will lead to death		
Yes	134	46.9
No	37	12.9
Never heard of cervical cancer	64	22.4
Do not know	48	16.8
Refused/missing	3	1.0
Believe your current doctor would also treat cervical cancer needs		
Yes	43	15.0
No	199	69.6
Do not know	28	9.8
Refused/missing	16	5.6
Believe vaccinations prevent disease		
Yes	237	82.9
No	13	4.5
Do not know	31	10.8
Refused/missing	5	1.8
“Very likely” or “likely” to be the person who makes decisions to vaccinate your child		
Yes	214	74.8
No	53	18.6
Refused/missing	19	6.6
Ever heard of HPV		
Yes	64	22.4
No	197	68.8
Do not know	20	7.0
Refused/missing	5	1.8
Know that HPV is main cause of cervical cancer		
Yes	34	11.9
No	61	21.3
Do not know	183	64.0
Refused/missing	8	2.8
Believe that your community might resist HPV vaccinations		
Yes	26	9.1
No	154	53.9
Do not know	85	29.7
Refused/missing	21	7.3

**Table 3:** Crude odds of having ever heard of HPV, Odisha, India (*n* = 286).

Characteristic	Crude odds ratio (95% confidence interval)
Age	
20–29 years	5.8 (2.4, 14.3)^*∗∗*^
30–39 years	3.0 (1.2, 7.5)^*∗*^
≥40 years	Reference
Marital status (single versus married/formerly married)	3.0 (1.6, 5.6)^*∗∗*^
Sexual history	
Never had sex	1.5 (0.8, 3.1)^NS^
First sex at <21 years	0.1 (0.0, 0.3)^*∗∗*^
First sex at ≥21 years	Reference

*∗* = <0.05; *∗∗* = <0.01; NS = not significant.
